# Relation of serum uric acid with the risk of coronary heart disease: an updated systematic review and dose-response meta-analysis of epidemiologic studies

**DOI:** 10.3389/fendo.2026.1762413

**Published:** 2026-02-20

**Authors:** Zhen Zhou, Guancheng Ye, Xiaoqiao Ren, Yaxing Cheng, Yuzhen Cang, Jingbo Zhao, Huihui Zhao

**Affiliations:** 1 School of Traditional Chinese Medicine, Beijing University of Chinese Medicine, Beijing, China; 2 Department of Rheumatology, Dongzhimen Hospital, Beijing University of Chinese Medicine, Beijing, China

**Keywords:** coronary heart disease, dose-response meta-analysis, meta-analysis, serum uric acid, systematic review

## Abstract

**Background:**

Over the past decade, multiple recent studies have investigated the relationship between serum uric acid(SUA) levels and the risk of coronary heart disease (CHD). This meta-analysis aims to assess the dose-response relationship between SUA levels and CHD by integrating the latest relevant studies from the past decade.

**Methods:**

Epidemiological studies such as cohort studies, which aim to explore the association between SUA levels and the risk of CHD, were included. Papers unrelated to the aforementioned topics, non-original works or studies, were not conducted with humans, were not published between 2015 to 2025 or that could not provide the specific numerical data required for analysis were excluded. We systematically searched the PubMed, Web of Science, EMBASE, and Cochrane Library databases until September 2025 for relevant studies. We performed a systematic review of relevant original studies and conducted both overall analysis and dose-response analysis. Data were pooled using a random-effects model, heterogeneity was assessed with the I² statistic, and the robustness of findings was evaluated via model comparison and sequential exclusion.

**Results:**

8 cohort studies were included. Among 574, 815 participants, there were 11,009 cases of CHD. An increased risk of CHD was associated with elevated SUA (HR = 1.38 95% CI = 1.30–1.47) in the overall meta-analysis. In the subgroup analysis, a consistent positive correlation was observed between SUA and the risk of CHD in all the pre-defined subgroups (including gender, sample size, publication year and outcome type). The dose-response meta-analysis demonstrated that elevated SUA levels were associated with increased CHD risk in a dose-dependent manner, with a non-linear pattern in males and a linear pattern in females.

**Conclusion:**

Dose-response analysis showed a positive, non-linear association between SUA and CHD in males, and a linear association in females, highlighting its role as a risk marker. Definitive evidence for causality requires future randomized controlled trials.

**Systematic Review Registration:**

https://www.crd.york.ac.uk/PROSPERO/recorddashboard, identifier CRD420251240886.

## Introduction

Coronary heart disease (CHD) is a leading cause of death and one of the most common chronic non-communicable diseases worldwide (1). Studies have shown that the prevalence of CHD has been consistently high across the globe over the past decades (2–4), and is even rising rapidly in developing countries (5, 6). For example, all studies conducted in the last two decades in India indicate a two to four fold rise in the prevalence of CHD (7), and data from China’s fifth health services survey in 2013 showed that the prevalence of CHD among Chinese residents had risen to 10.2%, and the prevalence of CHD among people over 60 years of age had even reached 27.8% (8). Such a high prevalence rate has brought heavy economic burdens and physical and mental damage to CHD patients and their families. Thus, exploring risk factors, early diagnosis, and early treatment plays a significant role in preventing and treating CHD. The aetiology of CHD currently remains unclear. A significant amount of research has reported on the risk factors for CHD, which include age, male gender, family history, hypertension, diabetes, obesity, hyperlipidaemia, smoking and lack of physical activity (9–17).

Accumulating research evidence has implied a potential connection between elevated serum uric acid (SUA) levels and the onset of coronary heart disease (CHD) (18). However, a definitive consensus has not been reached. For instance, certain studies indicate that elevated SUA has no significant value in predicting CHD risk (19), while others point to a robust association that persists even after adjusting for potential confounders (20). Furthermore, specific research suggests that elevated SUA is independently linked to coronary artery calcification (21). These inconsistent conclusions highlight the necessity for a dose-response meta-analysis to quantify the relationship between SUA levels and CHD risk.

Although a previous dose-response meta-analysis summarized the evidence up to 2015 (22), an updated synthesis incorporating more recent studies is warranted. This study aims to provide an updated synthesis of the evidence, with dedicated analyses to investigate non-linear patterns and potential differences by sex.

## Methods

### Search strategy

The PubMed, Web of Science, Cochrane Library, and Embase databases were searched for relevant articles published by September 2025, with the detailed search strategy available in **Supplementary Table S1**. This study also referred to the reference list of published papers and literature reviews to supplement other related research.

Our study has been registered in the International Prospective Register of Systematic Reviews (PROSPERO). (https://www.crd.york.ac.uk/PROSPERO/recorddashboard, identifier CRD420251240886). As detailed in **Supplementary Table S3** and **Supplementary Table S4**, this meta-analysis was conducted in accordance with the Preferred Reporting Items for Systematic Reviews and Meta-Analyses (PRISMA) statement (23).

### Selection criteria and study selection

The inclusion criteria were as follows: (1) participants: adult; (2) comparison: different levels of SUA; (3) outcomes: evaluated the relationship between the SUA levels and the risk of CHD; (4) types of studies: epidemiological studies; (5) reports of the hazard ratio (HR) estimates with the corresponding 95% confidence intervals (CIs) or having sufficient information to calculate them and provided useful data for the dose−response analysis; and (6) having clear sample size information.

The exclusion criteria were: (1) they were reviews, meta-analyses, case reports, case series, commentaries, letters and congress abstracts; (2) they were not conducted with humans; (3) no hazard ratio were reported or could not be extracted; (4) they were not related to SUA or CHD; (5) they were not published between 2015 to 2025; and (6) they used languages other than English.

Two researchers independently carried out the full process of our study from the screening for included studies to data analysis. The final articles included were selected by reviewing the title, abstract, and full text, with any disagreements resolved through discussion or by involving a third author if needed. When the article was unavailable or more information was needed for the analyses, an email was sent to the author requesting the article or additional details. The identification of duplicated manuscripts was done manually.

### Data extraction

Data from all eligible studies were meticulously extracted by two authors based on the inclusion criteria. If two authors could not reach a consensus, a third author was consulted to evaluate the article. The following data were extracted: name of first author, year of publication, country, study design, number of cases and total population, gender, outcome type, SUA levels, variables of adjustments, hazard ratio (HR) with 95% confidence interval (CI) from the most adjusted model.

### Risk of bias assessment

The risk of bias in the included articles was assessed by two authors utilizing Newcastle-Ottawa Scale (NOS) (24), disagreements were discussed and solved by the third author. The NOS rates stars based on three main criteria: selection (maximum of 4 points), comparability (maximum of 2 points), and outcome(maximum of 3 points), with a total of 8 items. Each study was assigned a NOS score (0–9), with a score of 7 or more indicating “high quality” and a score of less than 7 indicating “low quality”.

### Statistical methods

The strength of the association between SUA levels and the risk of CHD was assessed by using HR with 95% CI. Statistical heterogeneity was assessed using visual inspection of forest plots and statistical methods. The heterogeneity was evaluated by using the I² statistic, which can quantify the proportion of total variation among studies that is caused by heterogeneity rather than by chance factors. Although it is generally believed that an I² value greater than 50% indicates significant heterogeneity, we acknowledge that this threshold varies depending on the research context (25). Therefore, we also employed the Q test (with p<0.05 indicating statistical significance) for a comprehensive assessment to quantify the dispersion of the true effect. Additionally, we conducted subgroup analysis to verify the primary sources of heterogeneity and evaluate the stability of the combined results. The subgroups were defined as follows: gender(male, female), sample size(≤3000, >3000) the publication year(before2020, after 2020) and outcome type(broader CHD, myocardial infarction only). A dose-response meta-analysis was conducted by performing a trend analysis on the aggregated dose-response data using the generalized least squares trend estimation method. A linear relationship between SUA levels and the risk of CHD was initially assumed. Then, dose-response analyses for each study were performed using the glst command, and all dose-response data were pooled using restricted maximum likelihood. Publication bias was not assessed because fewer than ten studies were included in the meta-analysis, which renders statistical tests (e.g., Egger’s test) underpowered and unreliable. To evaluate the robustness of the pooled results, sensitivity analyses were performed using two complementary methods: first, a comparison of statistical models, in which the random-effects model was compared with a fixed-effects model to see if the results changed based on how we handled study differences; second, sequential exclusion of any single study method was used to assess its disproportionate influence on the overall estimate. Stata 12.0 was used for each statistical analysis(Stata Corp, College Station, Texas).

## Results

### Literature search and selection result

**Figure 1:**
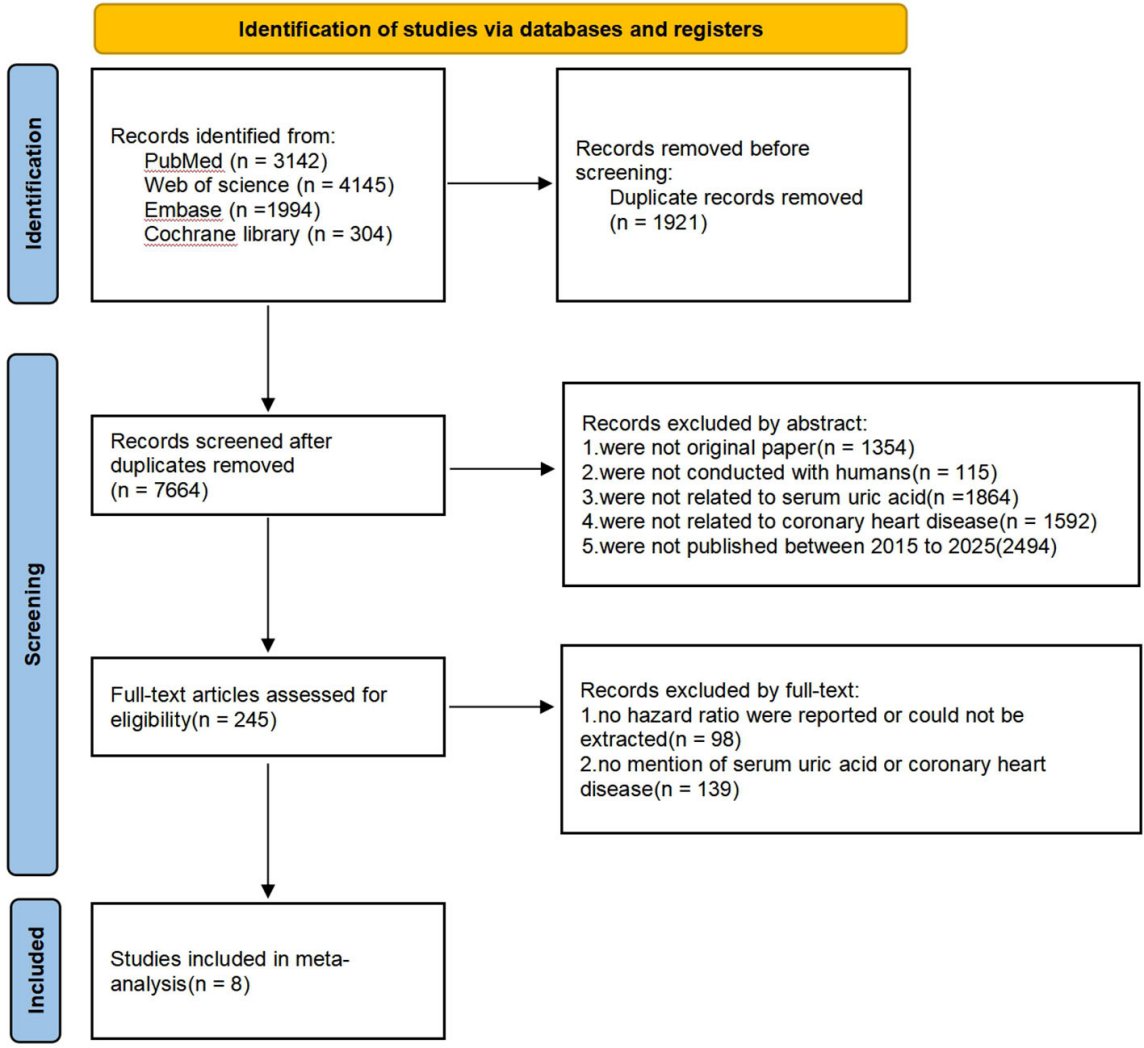
Flow diagram of the study search and selection process.

A flow chart was developed to illustrate the literature search and screening process (**Figure 1**). Based on the established search strategy, we conducted a search in four databases: PubMed, Web of Science, Cochrane Library, and Embase. A total of 9,585 articles were retrieved. After eliminating duplicate literature, 7,664 articles remained. According to the established inclusion and exclusion criteria, 7,419 articles were excluded after screening the titles and abstracts. Subsequently, 237 articles were further excluded after reading the full texts of the remaining documents. Finally, 8 cohort studies were included in this meta-analysis.

### Study characteristics and quality

The main characteristics of the included studies are shown in **Table 1**. 8 cohort studies with 11, 009 CHD cases and 574, 815 participants were included in this meta-analysis. In general, the sample sizes of the included studies ranged from 203 to 63,014, while the publication year ranged from 2016 to 2023. Among these articles, one of them was from Europe, while the others were all from Asia(China).

**Table 1:** Overview of included studies.

Author	Year	Location	Study design	Gender	Outcome type	Cases(n)	Total(n)		SUA levels(μ mol/L)	HR (95% CI)	Covaiates adjusted in models
Lai et al. (26)	2016	China	Cohort Study	All	Broader CHD	307	4033		166.1-243.1	1	A
				All		403	4019		228.4-292.0	1.23 (1.06-1.43)	A
				All		409	3994		267.8-340.6	1.13 (0.97-1.31)	A
				All		541	4107		321.2-453.0	1.26 (1.09-1.47)	A
Tian et al. (27)	2020	China	Cohort Study	All	Myocardial infarction only	707	63014		271.3	1	B
				All		46	2043		480.9	1.4 (1.02-1.92)	B
				Male		645	48856		<420	1	B
				Male		39	1718		≥420	1.34(0.96-1.87)	B
				Female		62	14158		<360	1	B
				Female		7	325		≥360	1.87(0.82-4.26)	B
Cheng et al. (28)	2021	China	Cohort Study	Male	Broader CHD	337	15733		≤420	1	C
				Male		16	626		421-434	0.98 (0.65-1.5)	C
				Male		23	697		435-455	1.2 (0.84-1.72)	C
				Male		19	687		456-487	0.93 (0.63-1.38)	C
				Male		41	688		>487	1.94 (1.47-5.26)	C
				Female		216	10603		≤360	1	C
				Female		10	203		360-372	1.38 (0.81-2.36)	C
				Female		19	246		373-392	1.8 (1.21-2.7)	C
				Female		7	244		393-422	0.67 (0.35-1.27)	C
				Female		13	247		>422	1.71 (1.03-2.35)	C
Mannarino et al. (29)	2021	Finland, France, Italy, The Netherlands, and Sweden	Cohort Study	Male	Broader CHD	14	427		295-336	1	D
				Male		8	434		336-385	2.48 (0.64-9.57)	D
				Male		15	426		>385	4.39 (1.22-15.8)	D
				Female		5	475		244-284	1	D
				Female		13	468		284-328	2.42 (0.85-6.92)	D
				Female		10	470		>328	1.5 (0.46-4.93)	D
Tian et al. (30)	2021	China	Cohort Study	All	Myocardial infarction only	73	13365		<305.89	1	E
				All		100	13365		305.89-371.03	1.16 (0.86-1.57)	E
				All		141	13365		371.03–452.47	1.47 (1.1-1.97)	E
				All		162	13365		≥452.47	1.49 (1.11-2)	E
				Male		46	8619		<305.89	1	E
				Male		71	9209		305.89-371.03	1.02(0.73-1.40)	E
				Male		122	11606		371.03–452.47	1.43(1.06-1.93)	E
				Male		137	9719		≥452.47	1.33(0.98-1.81)	E
				Female		27	4746		<305.89	1	E
				Female		29	4156		305.89-371.03	1.04(0.32-3.39)	E
				Female		19	1759		371.03–452.47	2.60(1.06-6.39)	E
				Female		25	3646		≥452.47	3.85(1.44-10.28)	E
Tian et al. (31)	2022	China	Cohort Study	All	Myocardial infarction only	342	44,124		183-289	1	F
				All		372	34,431		266-382	1.1 (0.95-1.28)	F
				All		103	6948		351-499	1.35 (1.07-1.71)	F
				Male		229	29615		183-289	1	F
				Male		329	30523		266-382	1.09(0.93-1.28)	F
				Male		99	6670		351-499	1.37(1.09-1.74)	F
				Female		113	14509		183-289	1	F
				Female		43	3908		266-382	1.15(0.66-2.03)	F
				Female		4	278		351-499	1.36(1.05-2.75)	F
Hu et al. (32)	2023	China	Cohort Study	All	Broader CHD	212	8437		≤265	1	G
				All		317	8529		266-319	1.26 (1.08-1.54)	G
				All		385	8337		320-376	1.51 (1.26-1.8)	G
				All		424	8387		≥377	1.62 (1.35-1.9)	G
				Male		217	5171		≤310	1	G
				Male		200	5038		311-354	1.04(0.86,1.26)	G
				Male		250	5038		355-404	1.35(1.13,1.62)	G
				Male		268	5058		≥405	1.39(1.16,1.67)	G
				Female		60	3377		≤231	1	G
				Female		81	3392		232-266	1.16(0.83,1.62)	G
				Female		113	3280		267-306	1.55(1.13,2.13)	G
				Female		149	3336		≥307	1.53(1.13,2.07)	G
Tian et al. (33)	2023	China	Cohort Study	All	Myocardial infarction only	270	8691		180–239	1	H
				All		411	10142		240–299	1.05 (0.8-1.37)	H
				All		326	6451		300–359	1.4 (1.08-1.82)	H
				Male		127	4085		180–239	1	H
				Male		271	6686		240–299	1.14(0.96-1.37)	H
				Male		103	5230		300–359	1.30(1.07-1.56)	H
				Female		143	4606		180–239	1	H
				Female		140	3456		240–299	1.01(0.73-1.40)	H
				Female		24	1221		300–359	1.31(0.88-1.97)	H

SUA, Serum Uric Acid; HR, Hazard Ratio; CI, Confidence Interval; A, age, gender, BMI, smoking, drinking, physical activity, education levels, hypertension, diabetes, hyperlipidemia, family history of CHD, eGFR and usage of diuretics, diet frequency categories except for the stratified covariate itself; B, age, gender, education, current smoker, current alcohol, physical activity, body mass index, fasting blood glucose, systolic blood pressure, diastolic blood pressure, hypertension, diabetes mellitus, dyslipidemia, antihypertensive medication, antidiabetic medication, lipid-lowering medication estimated glomerular filtration and C-reactive protein; C: age, gender, BMIs, physical exercise, smoking index, pack-year, lifetime total alcohol intake, kilogram-year, diabetes status, hypertension stage, triglyceride, high-density lipoprotein and low-density lipoprotein; D: age, sex, body mass index, waist-to-hip ratio, systolic blood pressure, diastolic blood pressure, diabetes mellitus presence, smoking status, triglyceride, low-density lipoprotein cholesterol, high-density lipoprotein cholesterol, glomerular filtration rate, alcohol intake, meat consumption, treatment with diuretics, antiplatelet, antidiabetic, lipid-lowering, antihypertensive drugs, allopurinol, colchicine and site of enrolment; **E**: age, sex,education, income, smoking status, drinking status, physical activity, history of hypertension, diabetes mellitus, and dyslipidemia,body mass index, systolic blood pressure, diastolic blood pressure, fasting blood glucose,antihypertensive agents, diuretics, hypoglycemic agents, lipid-lowering agents, estimated,glomerular filtration rate, and hs-CRP; **F**: age, gender, baseline SUA, education, income, smoking status, alcohol use, physical activity, history of hypertension, diabetes and dyslipidemia, antihypertensive agents, hypoglycemic agents, lipid-lowering agents, body mass index, systolic blood pressure, diastolic blood pressure, fasting blood glucose, total cholesterol, estimated glomerular filtration rate, and high-sensitivity C-reactive protein; **G**: age, sex, education level, smoking index, alcohol index; **H**: age, sex,education, income, smoking status, drinking status, physical activity, body mass index, systolic blood pressure, diastolic blood pressure, fasting blood glucose, mean serum uric acid,history of hypertension, diabetes, and dyslipidemia, antihypertensive agents, hypoglycemic agents, lipid-lowering agents, estimated glomerular filtration rate, and high-sensitivity C-reactive protein.

The cohort studies were evaluated by NOS. All of the NOS scores of the included studies ranged from 8 to 9, with an average of 8.5, which indicated a good quality for this meta-analysis(**Supplementary Table S2**).

### Overall analyses

**Figure 2:**
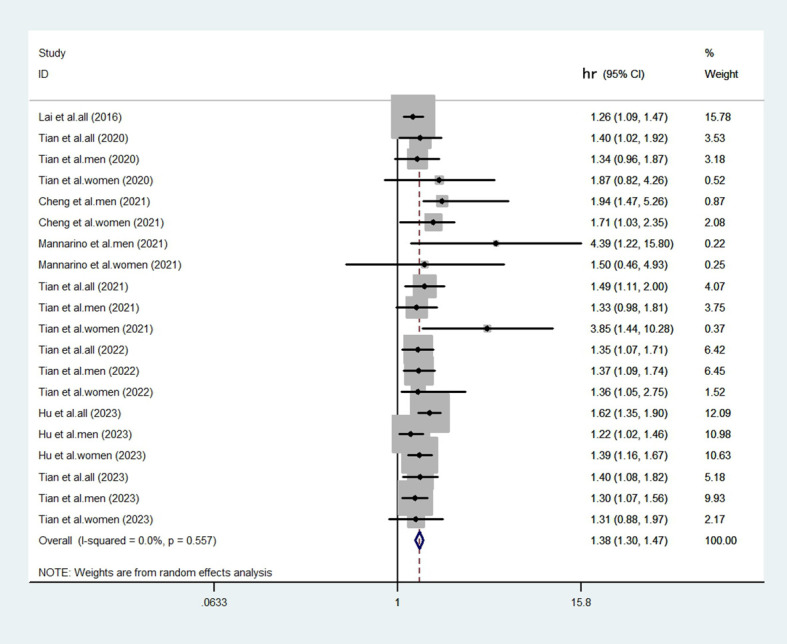
Forest plot generated by the random-effects model. The results showed that the elevated SUA was associated with an increased risk of CHD (HR = 1.38; 95% CI: 1.30-1.47).

**Figure 3:**
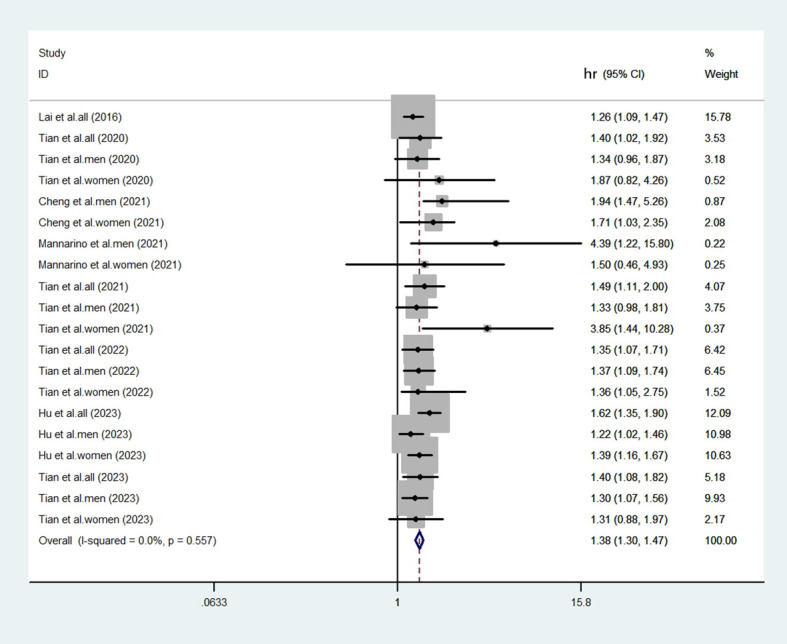
Forest plot generated by the fixed-effects model. The results showed that the elevated SUA was associated with an increased risk of CHD (HR = 1.38; 95% CI: 1.30-1.47).

The heterogeneity test revealed a low level of heterogeneity (I^2^ = 0.0%, p=0.557). Despite a low statistical heterogeneity, we employed a random-effects model for the primary analysis. This approach is more appropriate for observational epidemiology, as it accounts for inherent conceptual heterogeneity arising from differences in study design, population characteristics, and adjustment for confounders, which may persist even in the absence of significant statistical heterogeneity. The fixed-effects model results are also provided for comparison in a sensitivity analysis. We compared the highest and lowest levels of SUA to analyze the relationship between elevated SUA and the risk of CHD in eligible studies. After aggregating all the results (**Figures 2**, **3**), there was a statistically significant association between elevated SUA and the risk of CHD (HR = 1.38, 95% CI = 1.30-1.47).

### Subgroup analyses and sensitivity analysis

Subgroup analyses were carried out for the following variables: gender (male, female), sample size (≤3000, >3000), publication year(before 2020, after 2020) and outcome type (broader CHD, myocardial infarction only). Subgroup analyses by gender demonstrated a significant association between elevated SUA and CHD in males (HR = 1.32, 95% CI = 1.19-1.45) and females(HR = 1.46, 95% CI = 1.26-1.68) (**Supplementary Figure S1**). When stratified by sample size, both large sample subgroup (HR = 1.37, 95% CI = 1.28-1.47) and small sample subgroup (HR = 1.48, 95% CI = 1.26-1.73) revealed that elevated SUA increased the risk of CHD(**Supplementary Figure S2**). Analysis by publication year indicated a consistent association in studies published both before 2020 (HR = 1.30, 95% CI = 1.15-1.47) and after 2020 (HR = 1.41, 95% CI = 1.31-1.51) (**Supplementary Figure S3**). As for different outcome type, the summary HR for broader CHD was 1.41 (95% CI = 1.26-1.57), for the more specific outcome of myocardial infarction only, the pooled HR was 1.37 (95% CI = 1.25-1.50). Furthermore, no significant heterogeneity was observed across any of these subgroups.

**Figure 4:**
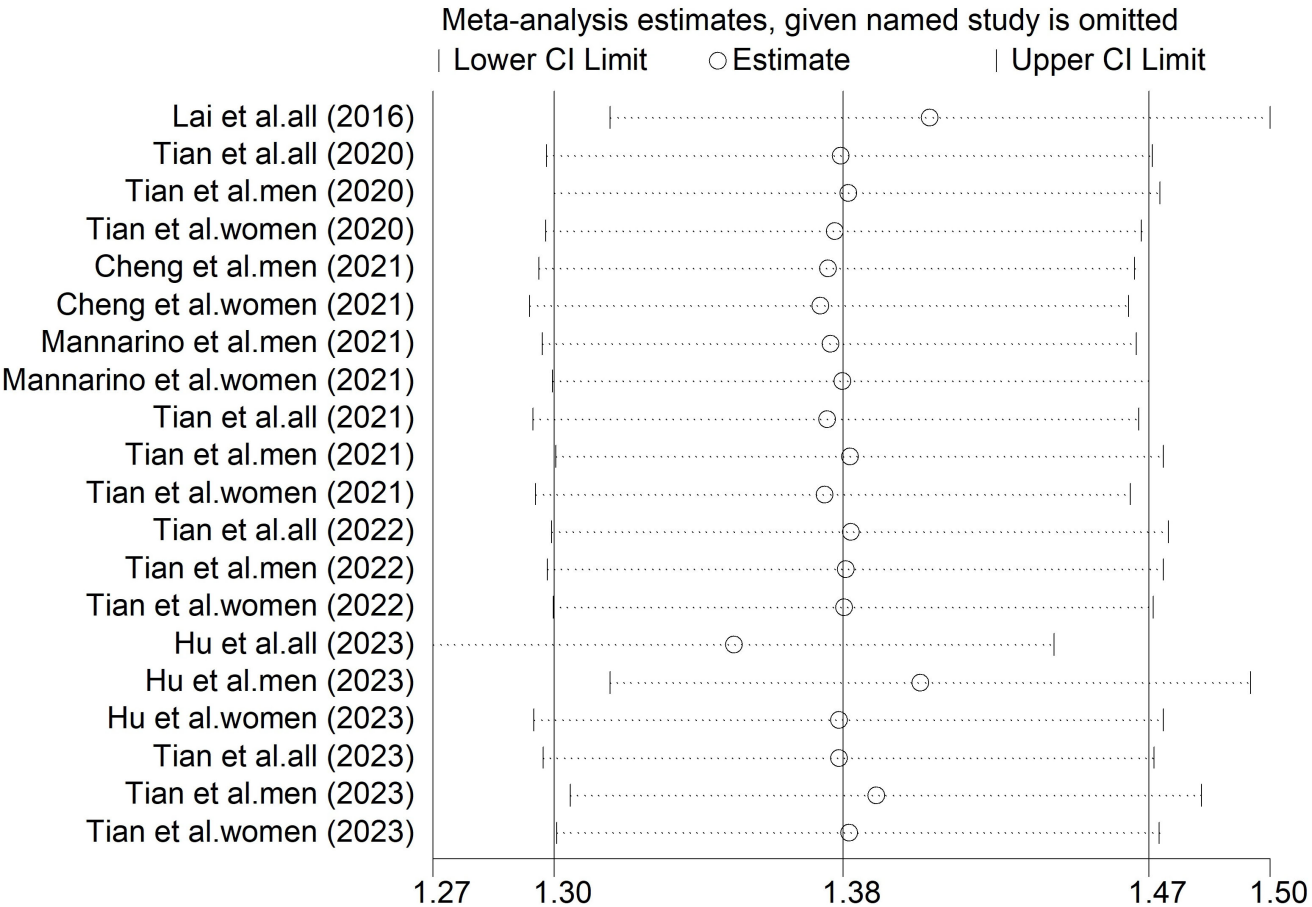
Sensitivity analysis diagrams for the studies assessing the association between elevated SUA and the risk of CHD.

The findings of the primary meta-analysis were robust across all pre-specified sensitivity analyses. The choice of meta-analytic model did not change the direction or statistical significance of the primary outcome. The pooled HR was 1.38 (95% CI = 1.30-1.47) under the random-effects model and 1.38 (95% CI = 1.30-1.47) under the fixed-effects model. The consistency between models strengthens confidence in the primary result (**Table 2**). In addition, sensitivity analysis using sequential exclusion of any single study method showed that the pooled effect estimate was not substantially altered by the removal of any single study (**Figure 4**).

**Table 2:** Sensitivity analysis: comparison of pooled effect estimates using random-effects and fixed-effects models.

Model​	No. of studies​	Pooled effect (95% CI)​	I²
Random-Effects	15	1.38 (95% CI = 1.30-1.47)	0.0%
Fixed-effects​	15	1.38 (95% CI = 1.30-1.47)	0.0%

### Dose-response analyses

**Figure 5:**
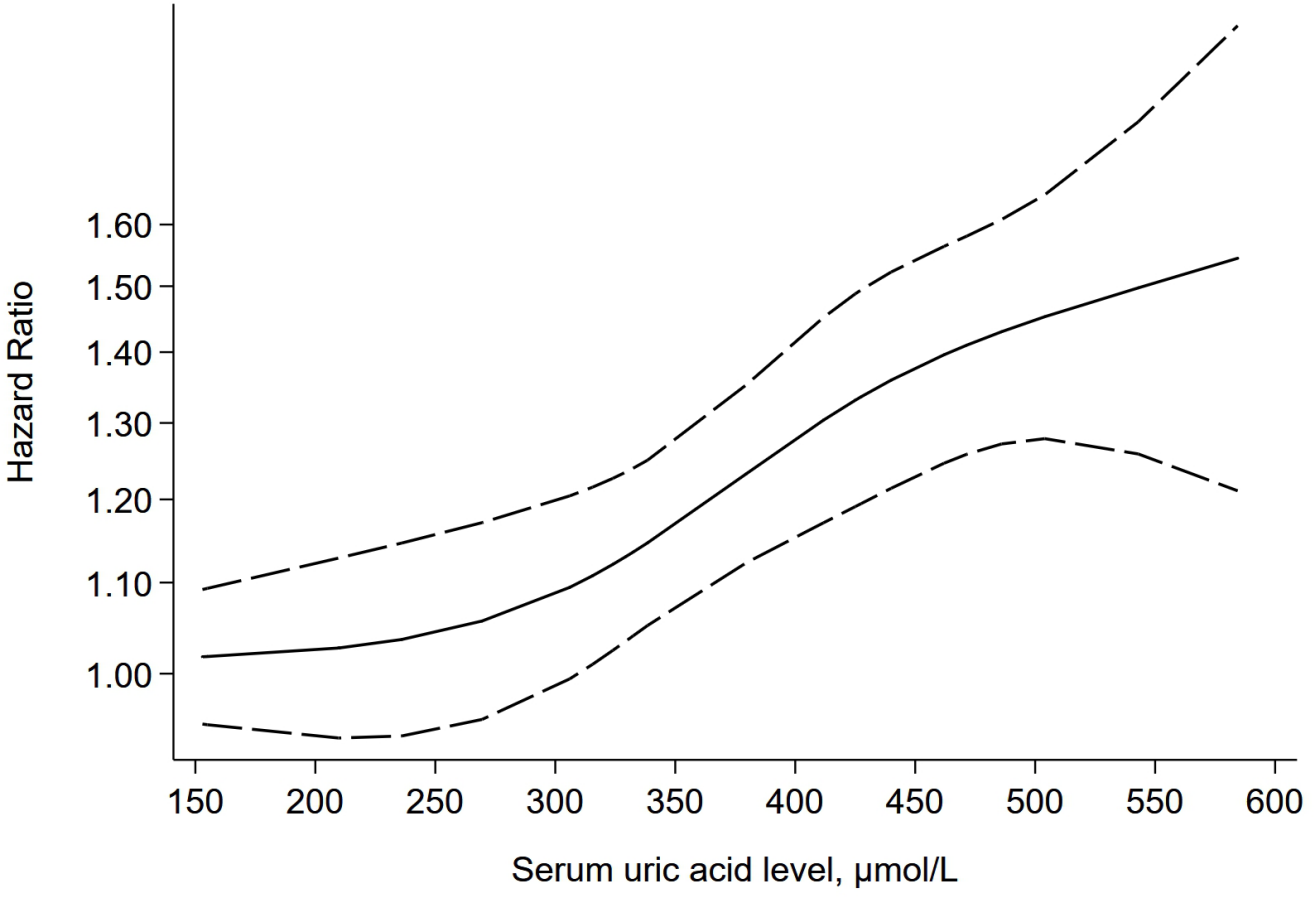
The dose-response plot on the association of different SUA levels and the risk of CHD in males.

**Figure 6:**
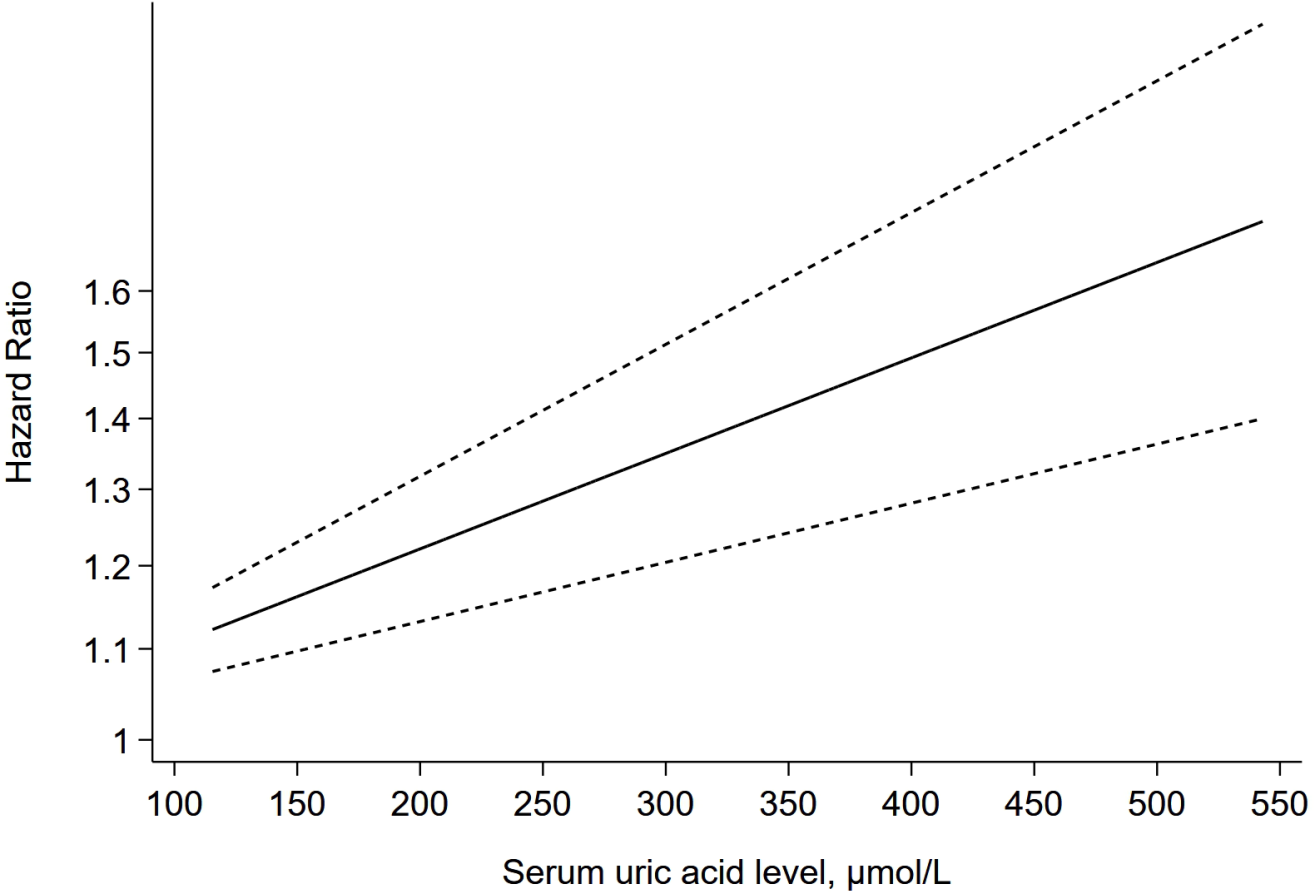
The dose-response plot on the association of different SUA levels and the risk of CHD in females.

7 studies (28–34) were further included in the dose-response meta-analysis to evaluate the dose-response relationship between different levels of SUA and the risk of CHD. Since the evaluation criteria for SUA differ between males and females, separate dose-response meta-analyses were conducted for males and females. The dose-response meta-analysis in males (**Figure 5**) indicated that there was a non-linear relationship between different SUA levels and the risk of CHD (P-non-linear < 0.05), and the higher the SUA level, the higher the risk of CHD. When the SUA level reached 324 μmol/L, the risk of CHD increased by 10% (HR = 1.10, 95% CI = 1.05-1.15, P < 0.05). The dose-response meta-analysis in females (**Figure 6**) suggested that there was a linear relationship between different SUA levels and the risk of CHD (P-linear < 0.05, P-non-linear > 0.05). The linear dose-response analysis found that each 94 μmol/L increase for SUA levels was associated with 10% higher CHD risk. The HR values at each SUA node for males and females are detailed in **Supplementary Tables S5**, **S6**.

## Discussion

CHD is associated with high rates of mortality and disability, posing a severe threat to human health. The purpose of this study is to assess the dose-response relationship between SUA levels and CHD by integrating the latest relevant studies from the past decade, the overall meta-analysis of 8 published epidemiological studies has shown that, compared with low level SUA, high level SUA leads to an increased risk of CHD. Dose-response meta-analysis has revealed a non-linear relationship between SUA levels and CHD risk in males, in contrast to a linear relationship in females, indicating a positive association in both sexes. A key and robust finding of our subgroup analysis is the consistent positive association between SUA and CHD risk across all pre-specified subgroup analyses, including gender, sample size, publication year and outcome type. The stability of this association across these diverse strata significantly strengthens the credibility of our primary conclusion that elevated SUA is an independent risk factor for CHD. The lack of a significant effect modification by gender suggests that the underlying pathophysiological mechanisms linking hyperuricemia to CHD-such as enhanced oxidative stress, systemic inflammation, and endothelial dysfunction-are likely operative in both men and women, underscoring the universal public health importance of SUA management. The consistent findings across studies of different sample sizes and publication year indicate that this relationship is not an artifact of a particular study design era or disproportionately influenced by a few large-scale investigations, thereby enhancing the generalizability of our results. Furthermore, the remarkably consistent risk estimates observed for both broader CHD (HR = 1.41) and myocardial infarction only (HR = 1.37), indicate that the positive association is robust and not an artifact of heterogeneous outcome type. In other words, elevated SUA may contribute to both the chronic atherosclerotic process and the acute thrombotic events culminating in MI. However, certain limitations must be considered. The consistency observed does not entirely rule out residual confounding by unmeasured factors, such as renal function or concomitant medications, at the individual level. Nevertheless, the remarkable consistency of our findings advocates for the consideration of SUA as a robust and generalizable biomarker for CHD risk stratification. Furthermore, our sensitivity analysis also indicates that the relationship between SUA and CHD risk is robust and reliable.

SUA, the end product of purine metabolism, is involved in various physiological and pathological processes. Consistent with our dose-response findings, elevated SUA levels are consistently and strongly associated with an increased risk of CHD in epidemiological studies (34, 35). The proposed pathogenic mechanisms linking hyperuricemia to CHD are multifaceted, primarily involving oxidative stress, endothelial dysfunction, inflammation, and vascular smooth muscle cell (VSMC) proliferation (18, 36). These pathways are hypothesized to interact synergistically to promote atherosclerosis. Experimental and observational evidence suggests that SUA can induce endothelial dysfunction by promoting oxidative stress and reducing nitric oxide bioavailability, a process considered a critical step in atherogenesis (37). It also acts as a pro-inflammatory stimulus, triggering cytokine release and sustaining a state of chronic vascular inflammation that may exacerbate plaque formation and vulnerability (38–40). Furthermore, SUA is shown to stimulate VSMC phenotypic switching and proliferation via pathways such as mitogen-activated protein kinase, thereby contributing to vascular remodeling and luminal narrowing (41, 42). The coherence of this mechanistic evidence supports the biological plausibility of the observed epidemiological association. This plausibility is further bolstered by observational data suggesting that UA-lowering therapy is associated with a reduced incidence of CHD (43), hypothetically through mitigating the aforementioned pathogenic pathways (44, 45).While the biological plausibility and observational data are compelling, it remains unclear whether lowering SUA per se translates to a decreased risk of incident CHD. This mirrors a pivotal debate in heart failure (HF) research, where hyperuricemia is a strong prognostic marker (e.g., with a risk threshold of 9.5 mg/dL). Importantly, randomized controlled trials (RCTs) in HF provide an important cautionary insight. The EXACT-HF trial showed that allopurinol significantly lowered SUA but did not improve clinical status, exercise capacity, or cardiac function in hyperuricemic HF patients (46). Similarly, the OPT-CHF trial found that oxypurinol failed to improve clinical outcomes in the overall cohort despite effectively reducing SUA (47). These trials demonstrate that while SUA is a robust risk marker and its lowering improves intermediate endpoints, this pharmacological intervention did not improve “hard” clinical outcomes in HF. This dissociation suggests that in cardiovascular contexts, elevated SUA may primarily be a marker of advanced disease severity and concomitant metabolic burden (e.g., insulin resistance, renal dysfunction) rather than a directly modifiable causal factor. Therefore, while our dose-response meta-analysis confirms a consistent and robust association between SUA and CHD risk, it cannot establish causality and does not constitute evidence for routine urate-lowering therapy for primary CHD prevention.​Definitive evidence must come from future, large-scale RCTs designed with primary cardiovascular endpoints to determine whether lowering SUA can prevent CHD events.

A key strength of this study is that it provides an updated dose-response meta-analysis on the relationship between SUA levels and CHD risk. By incorporating evidence published since the last meta-analysis, and by performing sex-specific dose-response analyses, our work refines the current understanding of this association. Our meta-analysis has the following limitations. First, there might be some unpublished data and research papers in non-English languages, and these factors could have influenced our research results. Second, only one of the studies meeting the inclusion criteria was conducted outside Asia. Therefore, further research is needed to explore the regional differences in the association between SUA and the risk of CHD. Third, as a meta-analysis of observational data, this study cannot establish causality and can only demonstrate an association between SUA and CHD risk. Last but not least, CHD predominates in middle-aged and elderly individuals, and in recent years, it has shown a trend of becoming more prevalent among younger people. Due to the limited information provided in the relevant research, this study was unable to conduct subgroup analysis by age. Further research is required to explore whether there are age-related and other demographic differences in the association between SUA and the risk of CHD.

## Conclusion

In conclusion, the overall meta-analysis indicate that individuals with elevated SUA may face a higher risk of CHD. Dose-response analysis demonstrated a positive association between SUA levels and CHD risk in both sexes, with the relationship being non-linear in males and linear in females. Based on this evidence, UA-lowering therapy may be associated with a reduced risk of CHD. While this association is consistent across subgroups and biologically plausible, evidence from analogous fields suggests SUA likely functions as a risk marker rather than a modifiable causal factor. Therefore, further research, particularly from randomized controlled trials, is needed to confirm these associations and evaluate any potential causal role.

## Data Availability

The original contributions presented in the study are included in the article/**Supplementary Material**. Further inquiries can be directed to the corresponding author.
