# Supplementary material for: Relation of serum uric acid with the risk of coronary heart disease: an updated systematic review and dose-response meta-analysis of epidemiologic studies
Source: Front Endocrinol (Lausanne). 2026 Feb 20;17:1762413. doi: 10.3389/fendo.2026.1762413 (PMC12963226; doi:10.3389/fendo.2026.1762413)
Supplement: Supplementary file 1 [file Table1.docx]

**Table S1** Search strategy: terms, databases and number of articles for review.

| **Databases** | **Strategy** | **Number of hits** | **Number imported into Endnote** |
| --- | --- | --- | --- |
| PubMed  Date (publication): Inception - 2025/9/14 | #1 Search (Coronary Disease[MeSH Terms]) OR (Angina Pectoris[MeSH Terms]) OR (Myocardial Infarction[MeSH Terms]) OR (Acute Coronary Syndrome[MeSH Terms]) OR (Myocardial Ischemia[MeSH Terms]) OR (Coronary Artery Disease[Title/Abstract]) OR (Coronary Heart Disease[Title/Abstract]) OR (CHD[Title/Abstract]) OR (Stenocardia[Title/Abstract]) OR (Angor Pectoris[Title/Abstract]) OR (Angina, Unstable[Title/Abstract]) OR (Angina, Stable[Title/Abstract]) OR (Angina[Title/Abstract]) OR (Myocardial Infarctions[Title/Abstract]) OR (Myocardial Infarct[Title/Abstract]) OR (Heart Attack[Title/Abstract]) OR (Acute Myocardial Infarction[Title/Abstract]) OR (Acute Coronary Syndromes[Title/Abstract]) OR (Coronary Syndrome, Acute[Title/Abstract]) OR (Myocardial Ischemias[Title/Abstract]) OR (Ischemic Heart Disease[Title/Abstract]) OR (Ischemic Heart[Title/Abstract])  #2 Search (Uric acid[MeSH Terms]) OR (2,6,8-Trihydroxypurine[Title/Abstract]) OR (Trioxopurine[Title/Abstract]) OR (Ammonium Acid Urate[Title/Abstract]) OR (Potassium Urate[Title/Abstract]) OR (Sodium Urate Monohydrate[Title/Abstract]) OR (Monosodium Urate[Title/Abstract]) OR (Monosodium Urate Monohydrate[Title/Abstract]) OR (Sodium Acid Urate[Title/Abstract]) OR (Sodium Acid Urate Monohydrate[Title/Abstract]) OR (Sodium Urate[Title/Abstract]) OR (Urate[Title/Abstract]) OR (Hyperuricemia[Title/Abstract]) OR (Gout[Title/Abstract]) OR (Gouts[Title/Abstract]) OR (Gouty Arthritis[Title/Abstract]) OR  (Gouty Arthritides[Title/Abstract])  #1 AND #2 | 626,882 51,310 | 3,132 |
| Web of Science  Date (publication): Inception - 2025/9/14 | #1 TS=(Coronary Disease OR Angina Pectoris OR Myocardial Infarction OR Acute Coronary Syndrome OR Myocardial Ischemia OR Coronary Artery Disease OR Coronary Heart Disease OR CHD OR Stenocardia OR Angor Pectoris OR Angina, Unstable OR Angina, Stable OR Angina OR Myocardial Infarctions OR Myocardial Infarct OR Heart Attack OR Acute Myocardial Infarction OR Acute Coronary Syndromes OR Coronary Syndrome, Acute OR Myocardial Ischemias OR Ischemic Heart Disease OR Ischemic Heart)  #2 TS=(Uric acid OR 2,6,8-Trihydroxypurine OR Trioxopurine OR Ammonium Acid Urate OR Potassium Urate OR Sodium Urate Monohydrate OR Monosodium Urate OR Monosodium Urate Monohydrate OR Sodium Acid Urate OR Sodium Acid Urate Monohydrate OR Sodium Urate OR  Urate OR Hyperuricemia OR Gout OR Gouts OR Gouty Arthritis OR Gouty Arthritides)  #1 AND #2 | 532,050  57,725 | 4,145 |
| EMBASE  Date (publication): Inception - 2025/9/14 | #1 'Coronary Disease':ab,ti OR 'Angina Pectoris':ab,ti OR 'Myocardial Infarction':ab,ti OR 'Acute Coronary Syndrome':ab,ti OR 'Myocardial Ischemia':ab,ti OR 'Coronary Artery Disease':ab,ti OR 'Coronary Heart Disease':ab,ti OR 'CHD':ab,ti OR 'Stenocardia':ab,ti OR 'Angor Pectoris':ab,ti OR 'Angina, Unstable':ab,ti OR 'Angina, Stable':ab,ti OR 'Angina':ab,ti OR 'Myocardial Infarctions':ab,ti OR 'Myocardial Infarct':ab,ti OR 'Heart Attack':ab,ti OR 'Acute Myocardial Infarction':ab,ti OR 'Acute Coronary Syndromes':ab,ti OR 'Coronary Syndrome, Acute':ab,ti OR 'Myocardial Ischemias':ab,ti OR 'Ischemic Heart Disease':ab,ti OR 'Ischemic Heart':ab,ti  #2 ‘Uric acid ’:ab,ti OR ‘2,6,8-Trihydroxypurine ’:ab,ti OR ‘Trioxopurine ’:ab,ti OR ‘Ammonium Acid Urate ’:ab,ti OR ‘Potassium Urate ’:ab,ti OR ‘Sodium Urate Monohydrate ’:ab,ti OR ‘Monosodium Urate ’:ab,ti OR ‘Monosodium Urate Monohydrate ’:ab,ti OR ‘Sodium Acid Urate ’:ab,ti OR ‘Sodium Acid Urate Monohydrate ’:ab,ti OR ‘Sodium Urate ’:ab,ti OR  ‘Urate ’:ab,ti OR ‘Hyperuricemia ’:ab,ti OR ‘Gout ’:ab,ti OR ‘Gouts ’:ab,ti OR ‘Gouty Arthritis ’:ab,ti OR ‘Gouty Arthritides ’:ab,ti  #1 AND #2 | 726,154  68,319 | 1,994 |
| Cochrane library  Date (publication): Inception - 2025/9/14 | #1 Coronary Disease OR Angina Pectoris OR Myocardial Infarction OR Acute Coronary Syndrome OR Myocardial Ischemia OR Coronary Artery Disease OR Coronary Heart Disease OR CHD OR Stenocardia OR Angor Pectoris OR Angina, Unstable OR Angina, Stable OR Angina OR Myocardial Infarctions OR Myocardial Infarct OR Heart Attack OR Acute Myocardial Infarction OR Acute Coronary Syndromes OR Coronary Syndrome, Acute OR Myocardial Ischemias OR Ischemic Heart Disease OR Ischemic Heart  #2 Uric acid OR 2,6,8-Trihydroxypurine OR Trioxopurine OR Ammonium Acid Urate OR Potassium Urate OR Sodium Urate Monohydrate OR Monosodium Urate OR Monosodium Urate Monohydrate OR Sodium Acid Urate OR Sodium Acid Urate Monohydrate OR Sodium Urate OR Urate OR  Hyperuricemia OR Gout OR Gouts OR Gouty Arthritis OR Gouty Arthritides  #1 AND #2 | 89,145  8,768 | 304 |
| **TOTAL RESULTS** |  |  | 9,585 |

**Table S2** Assessment of quality of included cohort studies by the Newcastle-Ottawa Scale.

| **First author, year** | **Selection** | | | | **Comparability** | | **Outcome** | | | **Total** |
| --- | --- | --- | --- | --- | --- | --- | --- | --- | --- | --- |
|  | **1** | **2** | **3** | **4** | **1** | | **1** | **2** | **3** |  |
| **Lai, 2016** | 1 | 1 | 1 | 1 | 1 | 1 | 1 | 1 | 1 | 9 |
| **Tian, 2020** | 1 | 1 | 1 | 1 | 1 | 1 | 1 | 1 | 0 | 8 |
| **Cheng, 2021** | 1 | 1 | 1 | 1 | 1 | 1 | 1 | 1 | 1 | 9 |
| **Mannarino, 2021** | 0 | 1 | 1 | 1 | 1 | 1 | 1 | 1 | 1 | 8 |
| **Tian, 2021** | 1 | 1 | 1 | 1 | 1 | 1 | 1 | 1 | 0 | 8 |
| **Tian, 2022** | 1 | 1 | 1 | 1 | 1 | 1 | 1 | 1 | 1 | 9 |
| **Hu, 2023** | 1 | 1 | 1 | 1 | 1 | 1 | 0 | 1 | 1 | 8 |
| **Tian, 2023** | 1 | 1 | 1 | 1 | 1 | 1 | 1 | 1 | 1 | 9 |

Note: A study can be awarded a maximum of one star for each numbered item within the Selection and Outcome categories. A maximum of two stars can be given for Comparability

Selection

1) Representativeness of the exposed cohort

a) truly representative of the average characteristics in the community ☆ 

b) somewhat representative of the average characteristics in the community ☆ 

c) selected group of users eg nurses, volunteers

d) no description of the derivation of the cohort

2) Selection of the non-exposed cohort

a) drawn from the same community as the exposed cohort ☆ 

b) drawn from a different source

c) no description of the derivation of the non-exposed cohort

3) Ascertainment of exposure

a) secure record ☆ 

b) structured interview ☆ 

c) written self-report

d) no description

4) Demonstration that outcome of interest was not present at start of study

a) yes ☆ 

b) no

Comparability

1) Comparability of cohorts on the basis of the design or analysis

a) study controls for the most important factor☆ 

b) study controls for any additional factor ☆

Outcome

1) Assessment of outcome

a) independent blind assessment ☆ 

b) record linkage ☆ 

c) self-report

d) no description

2) Was follow-up long enough for outcomes to occur

a) yes ☆ 

b) no

3) Adequacy of follow up of cohorts

a) complete follow up - all subjects accounted for ☆ 

b) subjects lost to follow up unlikely to introduce bias - small number lost - > 90% follow up, or description provided of those lost) ☆ 

c) follow up rate < 90% (select an adequate %) and no description of those lost

d) no statement

**Table S3** PRISMA_2020_checklist

| **Section and Topic** | **Item #** | **Checklist item** | **Location where item is reported** |
| --- | --- | --- | --- |
| **TITLE** | | |  |
| Title | 1 | Identify the report as a systematic review. | Pg1 |
| **ABSTRACT** | | |  |
| Abstract | 2 | See the PRISMA 2020 for Abstracts checklist. | Table S4 |
| **INTRODUCTION** | | |  |
| Rationale | 3 | Describe the rationale for the review in the context of existing knowledge. | Introduction(pg2) |
| Objectives | 4 | Provide an explicit statement of the objective(s) or question(s) the review addresses. | Introduction(pg2) |
| **METHODS** | | |  |
| Eligibility criteria | 5 | Specify the inclusion and exclusion criteria for the review and how studies were grouped for the syntheses. | Method(pg2-3) |
| Information sources | 6 | Specify all databases, registers, websites, organisations, reference lists and other sources searched or consulted to identify studies. Specify the date when each source was last searched or consulted. | Method(pg2) |
| Search strategy | 7 | Present the full search strategies for all databases, registers and websites, including any filters and limits used. | Figure1(pg2)  Table S1 |
| Selection process | 8 | Specify the methods used to decide whether a study met the inclusion criteria of the review, including how many reviewers screened each record and each report retrieved, whether they worked independently, and if applicable, details of automation tools used in the process. | Method(pg3) |
| Data collection process | 9 | Specify the methods used to collect data from reports, including how many reviewers collected data from each report, whether they worked independently, any processes for obtaining or confirming data from study investigators, and if applicable, details of automation tools used in the process. | Method(pg3) |
| Data items | 10a | List and define all outcomes for which data were sought. Specify whether all results that were compatible with each outcome domain in each study were sought (e.g. for all measures, time points, analyses), and if not, the methods used to decide which results to collect. | Method(pg3) |
|  | 10b | List and define all other variables for which data were sought (e.g. participant and intervention characteristics, funding sources). Describe any assumptions made about any missing or unclear information. | Method(pg3) |
| Study risk of bias assessment | 11 | Specify the methods used to assess risk of bias in the included studies, including details of the tool(s) used, how many reviewers assessed each study and whether they worked independently, and if applicable, details of automation tools used in the process. | Method(pg3) |
| Effect measures | 12 | Specify for each outcome the effect measure(s) (e.g. risk ratio, mean difference) used in the synthesis or presentation of results. | Method(pg2-3) |
| Synthesis methods | 13a | Describe the processes used to decide which studies were eligible for each synthesis (e.g. tabulating the study intervention characteristics and comparing against the planned groups for each synthesis (item #5)). | Method(pg2-3) |
|  | 13b | Describe any methods required to prepare the data for presentation or synthesis, such as handling of missing summary statistics, or data conversions. | Method(pg3) |
|  | 13c | Describe any methods used to tabulate or visually display results of individual studies and syntheses. | Method(pg3) |
|  | 13d | Describe any methods used to synthesize results and provide a rationale for the choice(s). If meta-analysis was performed, describe the model(s), method(s) to identify the presence and extent of statistical heterogeneity, and software package(s) used. | Method(pg3) |
|  | 13e | Describe any methods used to explore possible causes of heterogeneity among study results (e.g. subgroup analysis, meta-regression). | Method(pg3) |
|  | 13f | Describe any sensitivity analyses conducted to assess robustness of the synthesized results. | Method(pg3) |
| Reporting bias assessment | 14 | Describe any methods used to assess risk of bias due to missing results in a synthesis (arising from reporting biases). | Method(pg3) |
| Certainty assessment | 15 | Describe any methods used to assess certainty (or confidence) in the body of evidence for an outcome. | Method(pg3) |
| **RESULTS** | | |  |
| Study selection | 16a | Describe the results of the search and selection process, from the number of records identified in the search to the number of studies included in the review, ideally using a flow diagram. | Figure1(pg4) |
|  | 16b | Cite studies that might appear to meet the inclusion criteria, but which were excluded, and explain why they were excluded. | N/A |
| Study characteristics | 17 | Cite each included study and present its characteristics. | Results(pg4)  Table 1 |
| Risk of bias in studies | 18 | Present assessments of risk of bias for each included study. | Results(pg4) |
| Results of individual studies | 19 | For all outcomes, present, for each study: (a) summary statistics for each group (where appropriate) and (b) an effect estimate and its precision (e.g. confidence/credible interval), ideally using structured tables or plots. | Table 1 |
| Results of syntheses | 20a | For each synthesis, briefly summarise the characteristics and risk of bias among contributing studies. | Table 1  Table S2 |
|  | 20b | Present results of all statistical syntheses conducted. If meta-analysis was done, present for each the summary estimate and its precision (e.g. confidence/credible interval) and measures of statistical heterogeneity. If comparing groups, describe the direction of the effect. | Results(pg9) |
|  | 20c | Present results of all investigations of possible causes of heterogeneity among study results. | Results(pg9-10) |
|  | 20d | Present results of all sensitivity analyses conducted to assess the robustness of the synthesized results. | Results(pg10) |
| Reporting biases | 21 | Present assessments of risk of bias due to missing results (arising from reporting biases) for each synthesis assessed. | N/A |
| Certainty of evidence | 22 | Present assessments of certainty (or confidence) in the body of evidence for each outcome assessed. | Results(pg9-10) |
| **DISCUSSION** | | |  |
| Discussion | 23a | Provide a general interpretation of the results in the context of other evidence. | Discussion(pg11-12) |
|  | 23b | Discuss any limitations of the evidence included in the review. | Discussion(pg13) |
|  | 23c | Discuss any limitations of the review processes used. | Discussion(pg13) |
|  | 23d | Discuss implications of the results for practice, policy, and future research. | Conclusions and outlook(pg12-13) |
| **OTHER INFORMATION** | | |  |
| Registration and protocol | 24a | Provide registration information for the review, including register name and registration number, or state that the review was not registered. | pg1 |
|  | 24b | Indicate where the review protocol can be accessed, or state that a protocol was not prepared. | N/A |
|  | 24c | Describe and explain any amendments to information provided at registration or in the protocol. | N/A |
| Support | 25 | Describe sources of financial or non-financial support for the review, and the role of the funders or sponsors in the review. | pg14 |
| Competing interests | 26 | Declare any competing interests of review authors. | pg14 |
| Availability of data, code and other materials | 27 | Report which of the following are publicly available and where they can be found: template data collection forms; data extracted from included studies; data used for all analyses; analytic code; any other materials used in the review. | pg13 |

**Table S4** PRISMA 2020 for Abstracts Checklist

| **Section and Topic** | **Item #** | **Checklist item** | **Reported (Yes/No)** |
| --- | --- | --- | --- |
| **TITLE** | | |  |
| Title | 1 | Identify the report as a systematic review. | Yes |
| **BACKGROUND** | | |  |
| Objectives | 2 | Provide an explicit statement of the main objective(s) or question(s) the review addresses. | Yes |
| **METHODS** | | |  |
| Eligibility criteria | 3 | Specify the inclusion and exclusion criteria for the review. | Yes |
| Information sources | 4 | Specify the information sources (e.g. databases, registers) used to identify studies and the date when each was last searched. | Yes |
| Risk of bias | 5 | Specify the methods used to assess risk of bias in the included studies. | Yes |
| Synthesis of results | 6 | Specify the methods used to present and synthesise results. | Yes |
| **RESULTS** | | |  |
| Included studies | 7 | Give the total number of included studies and participants and summarise relevant characteristics of studies. | Yes |
| Synthesis of results | 8 | Present results for main outcomes, preferably indicating the number of included studies and participants for each. If meta-analysis was done, report the summary estimate and confidence/credible interval. If comparing groups, indicate the direction of the effect (i.e. which group is favoured). | Yes |
| **DISCUSSION** | | |  |
| Limitations of evidence | 9 | Provide a brief summary of the limitations of the evidence included in the review (e.g. study risk of bias, inconsistency and imprecision). | Yes |
| Interpretation | 10 | Provide a general interpretation of the results and important implications. | Yes |
| **OTHER** | | |  |
| Funding | 11 | Specify the primary source of funding for the review. | YES |
| Registration | 12 | Provide the register name and registration number. | YES |

**Table S5** The HR values at each SUA node for males

| **SUA node** | **Risk of CHD(HR)** | **95%CI** |
| --- | --- | --- |
| 152.95 | 1 | (1.00-1.00) |
| 155.00 | 1 | (1.00-1.00) |
| 209.50 | 1.01 | (0.99-1.03) |
| 210.00 | 1.01 | (0.99-1.03) |
| 236.00 | 1.02 | (0.99-1.05) |
| 269.50 | 1.04 | (1.00-1.07) |
| 306.00 | 1.08 | (1.04-1.12) |
| 315.50 | 1.09 | (1.05-1.13) |
| 324.00 | 1.1 | (1.05-1.15) |
| 329.50 | 1.11 | (1.06-1.17) |
| 332.50 | 1.12 | (1.06-1.17) |
| 338.46 | 1.13 | (1.07-1.19) |
| 379.50 | 1.21 | (1.10-1.34) |
| 411.75 | 1.28 | (1.13-1.46) |
| 425.00 | 1.31 | (1.14-1.50) |
| 427.50 | 1.31 | (1.15-1.50) |
| 440.00 | 1.34 | (1.16-1.53) |
| 462.00 | 1.37 | (1.20-1.57) |
| 471.50 | 1.39 | (1.21-1.58) |
| 486.00 | 1.41 | (1.23-1.61) |
| 504.00 | 1.43 | (1.25-1.64) |
| 542.96 | 1.47 | (1.25-1.74) |
| 584.40 | 1.52 | (1.21-1.90) |

**Table S6** The HR values at each SUA node for females

| **SUA node** | **Risk of CHD(HR)** | **95%CI** |
| --- | --- | --- |
| 115.50 | 1 | (1.00-1.00) |
| 152.95 | 1.04 | (1.02-1.05) |
| 180.00 | 1.07 | (1.04-1.09) |
| 209.50 | 1.1 | (1.06-1.14) |
| 236.00 | 1.13 | (1.08-1.18) |
| 249.00 | 1.14 | (1.09-1.20) |
| 264.00 | 1.16 | (1.10-1.23) |
| 269.50 | 1.17 | (1.10-1.24) |
| 286.50 | 1.19 | (1.11-1.27) |
| 306.00 | 1.21 | (1.13-1.30) |
| 324.00 | 1.23 | (1.14-1.33) |
| 329.50 | 1.24 | (1.14-1.34) |
| 338.46 | 1.25 | (1.15-1.36) |
| 366.50 | 1.29 | (1.17-1.41) |
| 368.40 | 1.29 | (1.17-1.42) |
| 382.50 | 1.31 | (1.18-1.45) |
| 393.60 | 1.32 | (1.19-1.47) |
| 407.50 | 1.34 | (1.20-1.50) |
| 411.75 | 1.34 | (1.20-1.51) |
| 425.00 | 1.36 | (1.21-1.53) |
| 432.00 | 1.37 | (1.22-1.55) |
| 506.40 | 1.48 | (1.27-1.72) |
| 542.96 | 1.53 | (1.30-1.80) |


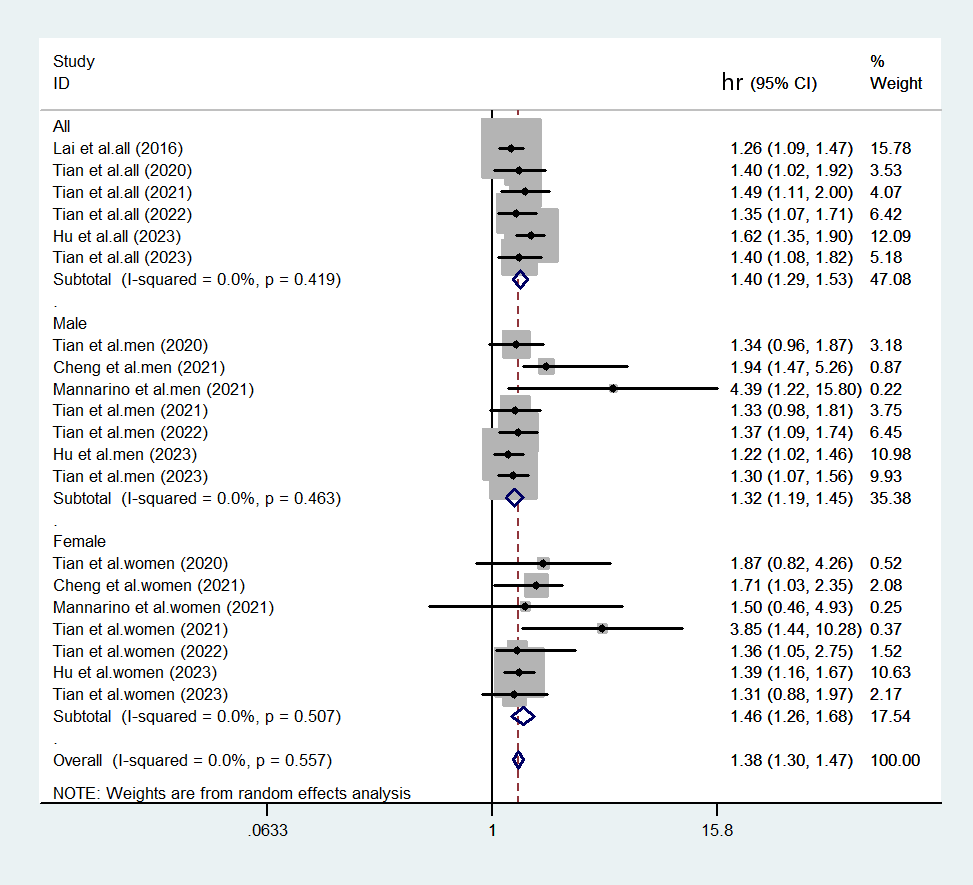


**Figure S1:** Summary of pooled HR with 95% CI for elevated SUA and the risk of CHD by gender


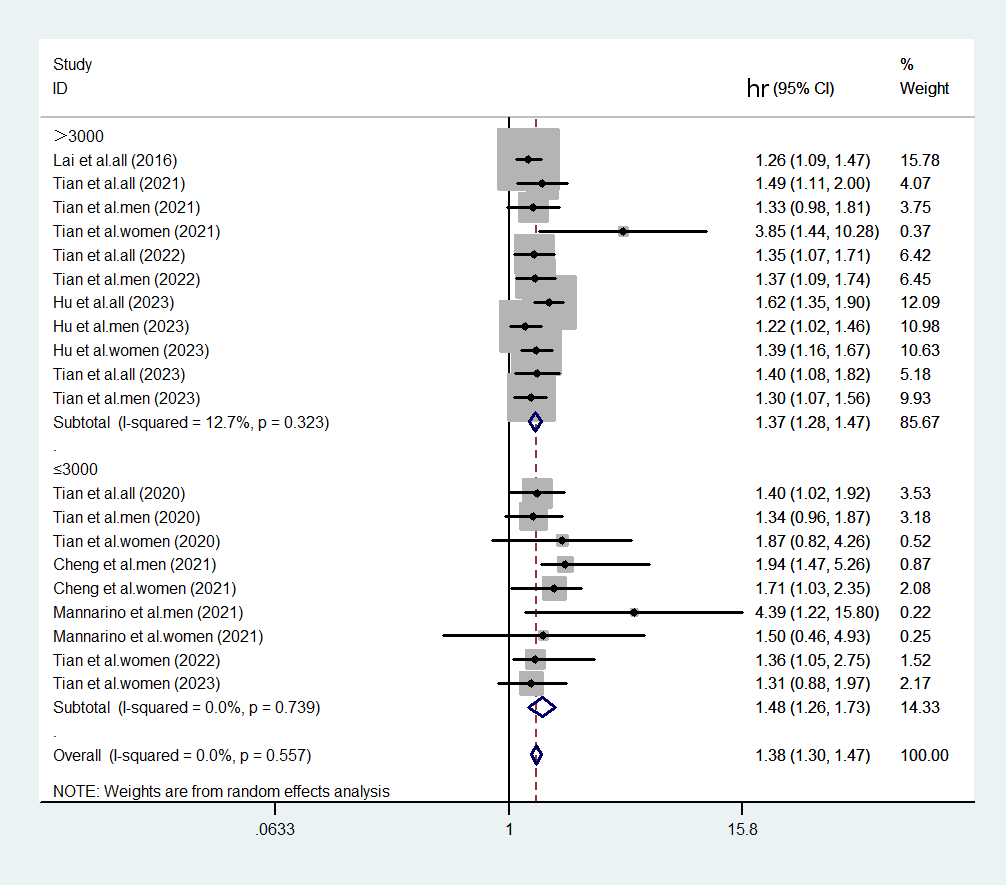


**Figure S2:** Summary of pooled HR with 95% CI for elevated SUA and the risk of CHD by sample size


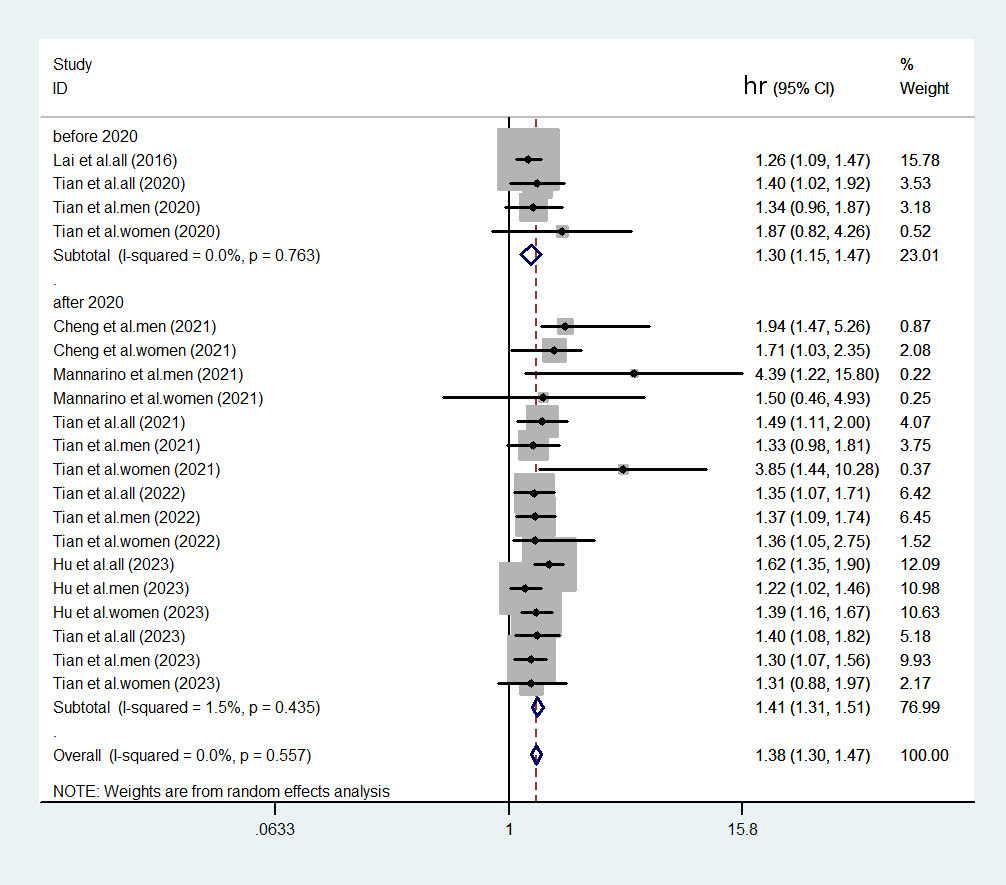


**Figure S3:** Summary of pooled HR with 95% CI for elevated SUA and the risk of CHD by publication year


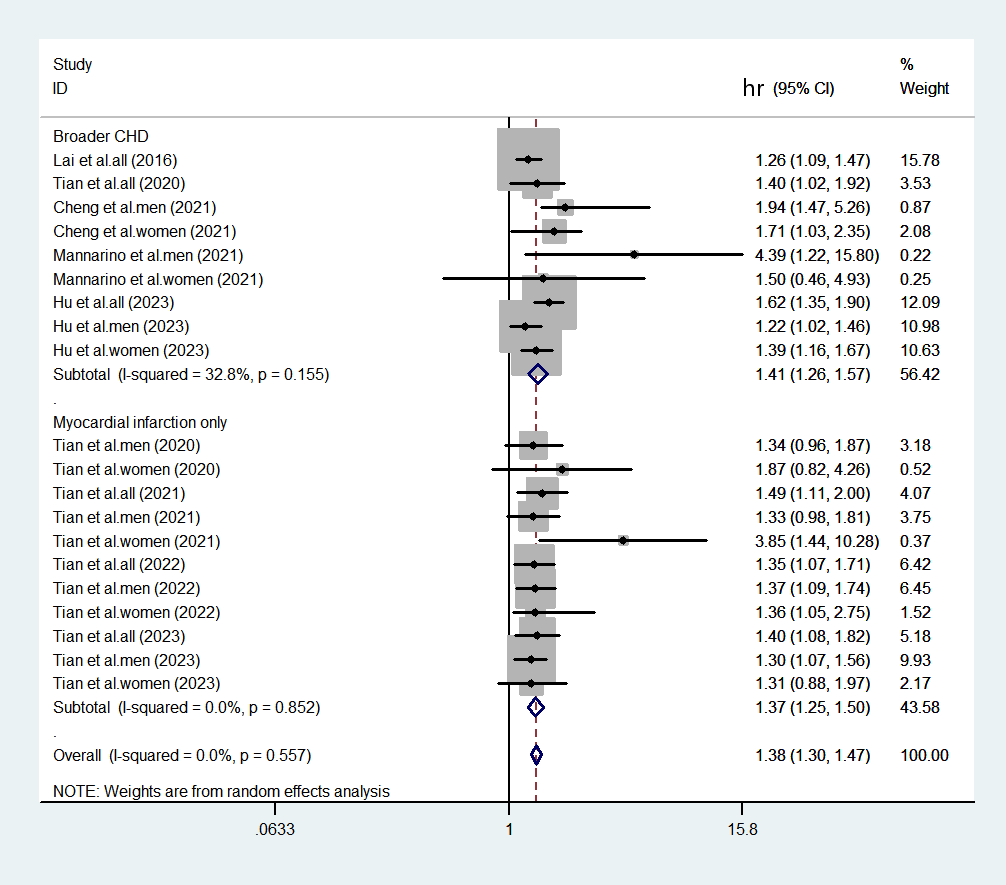


**Figure S4:** Summary of pooled HR with 95% CI for elevated SUA and the risk of CHD by outcome type
